# Digital Glide Path© for Fibre Post Retrieval Through Guided Endodontics in a Patient With Factor VIII Deficiency

**DOI:** 10.7759/cureus.117161

**Published:** 2026-09-29

**Authors:** Shikha Jaiswal, Sachin Gupta, Ayushi Sharma, Junaid Khan, Prachi Chaturvedi

**Affiliations:** 1 Department of Conservative Dentistry and Endodontics, Subharti Dental College and Hospital, Swami Vivekanand Subharti University, Meerut, IND

**Keywords:** cone-beam computed tomography, fibre post, guided endodontics, nonsurgical endodontics, post retrieval

## Abstract

Fibre posts are used in restorative dentistry to reinforce structurally weakened endodontically treated teeth that require additional retention to support the core. However, in cases of endodontic failure, retrieving posts is challenging with conventional endodontic retreatment procedures because the post adapts closely to the root canal walls and visibility is limited. Traditional freehand methods carry a risk of excessive dentin loss, root perforation and unpredictability. Guided endodontics, which utilises cone-beam computed tomography (CBCT) and a 3D-printed guide, offers a precise and minimally invasive alternative for post removal.

This case report illustrates the use of static guided endodontics utilising a Digital Glide Path^©^ for removal of a fibre post during nonsurgical endodontic retreatment of a maxillary lateral incisor (tooth 12) diagnosed with asymptomatic apical periodontitis in a 27-year-old patient with factor VIII deficiency. An intraoral periapical radiograph of 12 showed a fibre post, inadequate obturation of the apical third, and an associated periapical radiolucency. The patient’s blood report showed a 12% FVIII, 2BU/ml inhibitor and a prolonged APTT value, suggesting that an elective periapical surgery should not be undertaken without perioperative haemostatic control / Factor VIII replacement. Hence, a non-surgical guided endodontic approach was planned after obtaining written informed consent from the patient.

A DICOM file was acquired to visualize the spatial orientation of the fibre post within the root canal system. A surface scan of 12 was performed to generate an STL file, and planning software integrated both files to generate a Digital Glide Path^©^ for fibre post removal. A 3D resin template was fabricated to precisely guide a specialised bur to the apical extent of the fibre post, thereby facilitating its precise removal. Thus, Digital Glide Path^©^ minimised procedural errors in fibre post removal while serving as a predictable alternative to surgical retreatment in a hemophilic patient.

## Introduction

Endodontic retreatment of teeth with periapical lesions can be time-consuming and challenging. The goals are to gain access to the endodontic system to remove the entire root canal filling, perform chemo-mechanical disinfection, and promote apical healing [1,2]. If present, intra-radicular posts must be removed carefully to access the root canal filling in the apical part of the root. Fibre post removal should be atraumatic and directed towards maximizing radicular dentin preservation [3]. A thorough assessment of the factors influencing successful post removal is essential for predictable outcomes and ensuring the long-term success of nonsurgical endodontic retreatment. Fibre post retrieval is a technically demanding procedure, with its complexity largely influenced by factors such as the post type, luting cement, and residual dentin thickness. The process is often limited by the clinician’s experience, choice of technique, and inadequate visualisation. Unaided freehand post removal with conventional techniques utilising long-neck round burs is unpredictable, time-consuming, and often results in inadvertent dentin loss and/or perforation, eventually resulting in a guarded prognosis [4].

In such cases, static guided endodontics offers a conservative and predictable approach for fibre post retrieval while minimising unnecessary removal of sound tooth structure by creating a Digital Glide Path^©^ (Registration no. L-100840/2021, dated 24 March 2021; Copyright Office, Government of India), which is a digitally planned virtual drilling path. The workflow for preparing this path involves merging cone-beam computed tomography (CBCT) data in Digital Imaging and Communications in Medicine (DICOM) format with an optical surface scan in stereolithography (STL) format [5,6]. The virtual glide path is copied onto a 3-D printed guide(template), used intraorally to place a drill up to the targeted terminal point.

CBCT assessment is indicated when guided endodontic treatment is planned [7]; however, its use should be justified on an individual basis following clinical and two-dimensional radiographic assessment, with the smallest field of view appropriate for the diagnostic and treatment-planning task selected. Beyond post retrieval, this workflow has also been employed to establish controlled access paths in calcified root canals and to manage complex anatomical variations, such as Oehlers Type III dens invaginatus in maxillary lateral incisors [8].

For fibre-post retrieval, virtual planning permits assessment of the post axis, diameter, apical extent, surrounding dentine thickness, bur dimensions, target depth, and proposed point of entry before treatment. Clinical reports, comparative laboratory studies, and a recent systematic review indicate that static guidance can improve drilling accuracy, reduce operator dependence, and limit unnecessary dentine removal in selected cases. Nevertheless, it requires accurate DICOM-STL registration, stable guide support, adequate mouth opening, and a straight-line path that can be reached by the selected bur. These requirements mean that guided retrieval complements, rather than replaces, magnification and clinical judgment [9-12].

Medical comorbidity may further influence treatment selection. In haemophilia A, most restorative procedures are considered to have a relatively low bleeding risk, whereas surgical procedures require an individualized hemostatic plan developed with the hematology team. Decisions regarding factor replacement, antifibrinolytic therapy, and local haemostatic measures depend on the planned procedure, factor activity, bleeding phenotype, and inhibitor status [13]. The predictability and minimum invasiveness of guided endodontics in orthograde removal of fibre post establishes its superiority over the freehand technique and as a non-surgical alternative to apicoectomy, thus minimizing bleeding complications in hemophilic patients.

This report describes the use of a Digital Glide Path^©^ incorporated into a three-dimensional printed static guide for non-surgical removal of a fibre post from a maxillary lateral incisor in a patient with factor VIII deficiency.

## Case presentation

A 27-year-old man with congenital haemophilia A was referred for endodontic retreatment of tooth 12, which had previously received root canal treatment and a post-and-core restoration. Clinical examination revealed an asymptomatic tooth 12 restored with a composite core (Figure 1a). An intraoral periapical radiograph of the concerned tooth showed a fibre post, inadequate obturation of the apical third, and an associated periapical radiolucency (Figure 1b). A diagnosis of asymptomatic apical periodontitis was established. CBCT imaging showed the extent of periapical bone loss in relation to tooth 12 (Figure 1c). Because the patient had a known history of haemophilia A, we performed a comprehensive haemostatic evaluation, including factor VIII activity, activated partial thromboplastin time (aPTT), and factor VIII inhibitor titer using the Bethesda assay (Table 1). A factor VIII activity of 12%, the presence of a 2 BU/mL inhibitor, and a prolonged activated partial thromboplastin time (aPTT) indicated an increased risk of perioperative bleeding and necessitated appropriate haemostatic management. Therefore, we planned a minimally invasive, nonsurgical guided endodontic approach to avoid the additional surgical morbidity associated with conventional intervention [10].

**Table 1:** Haematological lab values. Interpretation: *aPTT is prolonged; *Factor VIII activity is decreased; *Bethesda inhibitor titer is positive. INR: International Normalized Ratio

Test Name	Results	Units	Bio Reference Interval
Hemoglobin	13.2	g/dL	13–17
Total leukocyte count	7,400	cells/µL	4,000–11,000
Platelet count	2.68	x10⁵/µL	1.5–4.5
INR	1.02		0.8–1.2
Activated partial thromboplastin time (aPTT)	52 ^*^	s	25–35
Factor VIII activity	0.12^*^(12%)	IU/mL	0.55–2.00 (55–200%)
Bethesda inhibitor titer	2^*^	BU/mL	>0.5 -0.6

Procedure

We created a Digital Glide Path^©^ by merging an STL surface scan with the DICOM dataset in digital planning software as follows.

Acquisition of the STL File

A diagnostic maxillary arch cast was produced, and the area of interest was digitized with an extraoral scanner (inEos X5, Dentsply Sirona). The surface scan was exported as an STL file.

Acquisition of the DICOM File

A high-resolution CBCT scan of the area of interest was acquired with a 0.1-mm voxel size and a 60 × 60-mm field of view using an Orthophos SL unit (software version V05.10.02; Dentsply Sirona). The data were exported in DICOM format for three-dimensional visualisation of tooth 12.

Digital Glide Path^©^ Creation

A 28-mm-long diamond bur with a 1.0-mm tip diameter and a 1.5-mm shank diameter was selected for use with the sleeved guide. The DICOM and STL datasets, along with a virtual representation of the selected bur, were imported into Exoplan software (exocad GmbH, Germany). The datasets were aligned and merged using a best-fit algorithm based on common reference landmarks (Figure 1d). A virtual three-dimensional guide incorporating a Digital Glide Path^©^ was designed to direct the bur to the apical end of the fibre post (Figure 1e). To guide the bur, a 5-mm-long metal sleeve with an internal diameter of 1.5 mm and an external diameter of 3.5 mm was incorporated into the virtual guide design over tooth 12. The virtual path and endpoint of fibre post removal were planned based on the fibre post’s location and apical extent.

The guide design was exported as an STL file and 3-D printed in resin (Formlabs Inc., Somerville, MA, USA)

Operative Procedure

The fit and stability of the guide were verified on the cast and intraorally (Figure 1f). The selected bur was mounted in a slow-speed contra-angle handpiece and advanced along the glide path through the sleeved template. The fibrepost was drilled using an interrupted pecking motion, advancing the drill in 1-mm increments. The post space was inspected under a dental operating microscope at ×15 magnification and irrigated with saline after each increment. Drilling continued until the targeted endpoint was reached and access to apical obturating material was obtained. Complete post removal was then done, which was confirmed microscopically (Figure 1g), radiographically and clinically(no more visible segments of residual fibre post or luting cement were obtained from the canal). The guide was then removed, and residual obturating material in the apical third was removed with H-files (M-Access, Dentsply) under rubber dam isolation. Working length was confirmed both electronically (Canal Pro AL, Coltene) and radiographically (Figure 1h). Since the initial file size after GP removal corresponded to a 50 K -file, the apical root canal was manually instrumented to size 80 K-file (M-Access, Dentsply) and irrigated using passive ultrasonic activation. Aqueous calcium hydroxide paste (UltraCal XS, Ultradent) was placed as an intracanal medicament for 2 weeks and repeated. At the end of the 4th week, the intracanal medicament was removed, and the apical 5mm of the root canal was obturated with mineral trioxide aggregate (MTA Angelus) to provide a superior, moisture-tolerant seal and to actively support the regeneration of damaged bone in the periapical tissues. One week later, the canal was reinforced with a fibre post-and-core restoration (Figure 1i). The patient deferred fabrication of a full-coverage crown while the retreatment outcome was monitored. Continued healing of the periapical lesion was observed in a subsequent recall visit at six months.

**Figure 1:**
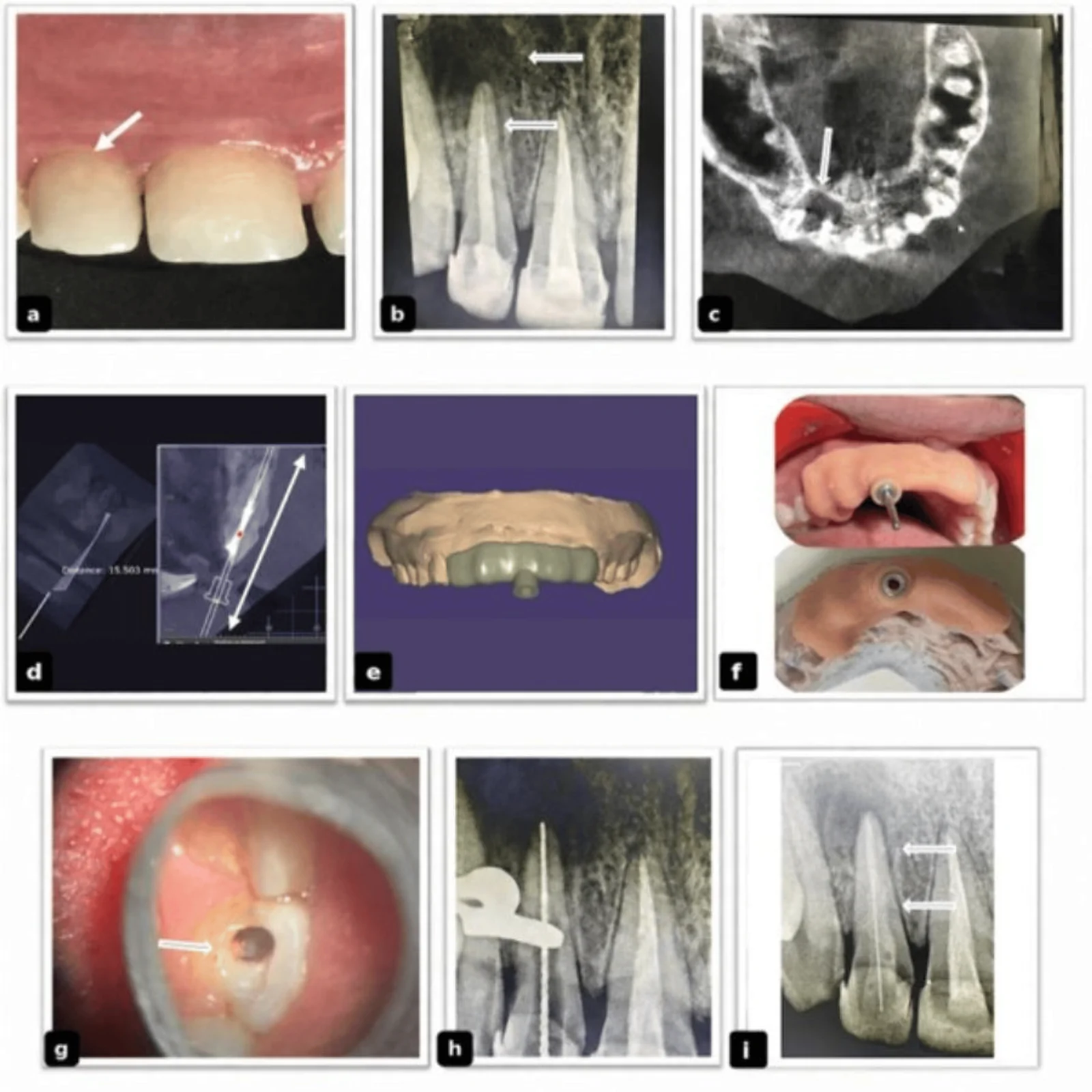
(a) Preoperative clinical photograph of post-and-core restored tooth 12; (b) Preoperative IOPA showing the fibre post, inadequate apical obturation and periapical radiolucency; (c) Axial CBCT image showing the extent of the periapical lesion; (d) Digital Glide Path image; (e) Virtual 3-D guide before printing; (f) 3-D printed guide test-fitted intraorally and extraorally; (g) Microscopic view showing post removal till targeted endpoint; (h) Working-length radiograph after canal patency was regained; (i) Postoperative radiograph (at 6-month follow-up) showing apical MTA obturation, bonded fibre post-and-core restoration and continued periapical healing.

## Discussion

Inadequate obturation of the apical root canal may permit persistent intraradicular infection and contribute to post-treatment apical periodontitis. When orthograde correction is feasible, nonsurgical retreatment permits renewed access, disinfection, and correction of the apical seal while avoiding surgical access. A bonded fibre post may be the first obstacle, and conventional burs or ultrasonic instruments can remove sound dentine when the post, luting cement, and root dentine are not clearly differentiated [4].

In haemophilia A, the choice among nonsurgical retreatment, endodontic surgery, and extraction must also account for the anticipated bleeding risk. Although the World Federation of Haemophilia classifies most restorative dental procedures as low risk, the use of statically guided digital glide-path preparation further supported a minimally invasive, nonsurgical orthograde approach, thereby providing a viable alternative to surgical intervention.

CBCT provided three-dimensional assessment of the post axis, apical extent, and residual dentine. The virtual bur was aligned with the post axis and positioned to terminate at the predetermined endpoint. Critical planning variables included accurate DICOM-STL registration, a stable tooth-supported guide, close sleeve-bur compatibility, sufficient bur length, an accessible point of entry, a defined target depth, and an adequate dentine safety margin. Scan artifacts, registration error, printing inaccuracy, sleeve clearance, incomplete guide seating, and intraoperative guide movement can each transfer error to the drilling path [6,9,14]. Hence, clinical verification of the guide before drilling becomes imperative.

Freehand and static guided post removal have their own pros and cons. A freehand approach is immediately available, cost-effective, and permits continuous modification of the drilling direction under magnification. Its success, however, depends heavily on operator experience and visual discrimination, particularly when a tooth-coloured post and resin cement blend with dentine. Static guidance constrains the bur to a predetermined trajectory and depth, which can make the initial approach more reproducible and precise. Conversely, it cannot be redirected readily if the clinical situation differs from the digital plan, and guide bulk, restricted irrigation, heat generation, debris accumulation, limited mouth opening, and residual cement or post material may complicate [4,14-16]

Direct comparative evidence supports a conservative advantage for static guidance, while also defining its limits. In an in vitro study of 90 human maxillary central incisors, Krug et al. [10] reported significantly less radicular dentin loss and a shorter working time with guided endodontic fibre-post removal compared with conventional techniques. Although procedural deviations were observed, no root perforations occurred in any of the groups. Abella Sans et al. found that static navigation reached the gutta-percha more frequently, required less time, and produced fewer perforations than freehand ultrasonic removal, with no significant performance difference between experienced and inexperienced operators [11]. A 2025 systematic review and meta-analysis of guided versus non-guided fiber-post removal found significantly greater accuracy with guided techniques, particularly in terms of angular and linear deviations. However, differences in procedure time and dentin loss were not statistically significant, and the available evidence was derived primarily from experimental studies, highlighting the need for further standardized clinical research [12]. These findings support technical precision and reliability but do not yet establish superior long-term clinical outcomes.

In the present case, the sleeved guide provided a controlled path to the apical extent of the fibre post, after which canal patency and access to the apical root filling were regained without an observed procedural complication. Microscopic inspection, staged drilling in 1-mm increments, and irrigation between increments remained essential to provide continuous visualization and debris removal, ensuring safety and minimizing procedural errors.

## Conclusions

Digital Glide Path^©^ with static-guided endodontics provides a minimally invasive and predictable approach to endodontic retreatment when posts obstruct complete access to the canal. It facilitates precise post removal while minimizing unnecessary dentine removal during complex nonsurgical retreatment.
